# Improved methods for magnetic purification of malaria parasites and haemozoin

**DOI:** 10.1186/1475-2875-9-17

**Published:** 2010-01-14

**Authors:** Charles C Kim, Emily B Wilson, Joseph L DeRisi

**Affiliations:** 1 Howard Hughes Medical Institute, Department of Biochemistry and Biophysics, University of California San Francisco, San Francisco, CA 94158, USA

## Abstract

**Background:**

Malaria parasites generate free haem upon catabolism of host haemoglobin during their intraerythrocytic growth cycle. In order to minimize oxidative toxicity of the ferric iron, the free haem molecules are polymerized into the biomineral beta-haematin (commonly referred to as haemozoin). Haemozoin crystals are paramagnetic, and this property can be exploited for the purification of late stage parasites as they contain larger haemozoin crystals than early stage parasites and uninfected cells. Commercially available magnets that were originally developed for the purpose of antibody-mediated cell purification are widely used for this purpose. As these methods are not necessarily optimized for parasite purification, the relationship between magnetic field strength and the quantity and quality of yield during parasite purification was explored.

**Methods:**

Inexpensive rare-earth neodymium magnets with commercially available disposable columns were employed to explore the relationship between magnetic field strength and recovery of free haemozoin and infected erythrocytes (iRBCs).

**Results:**

Yields of free haemozoin increased nearly linearly with increasing magnetic field strength to the strongest fields tested (8,500 Gauss). Stronger magnetic fields also improved the recovery of iRBCs with no detrimental effects on parasite viability. An in-house constructed magnetic stand, built for $75 in materials, produced superior results when compared with much more expensive commercial products.

**Conclusions:**

Existing protocols for the magnetic purification of free haemozoin and iRBCs result in sub-optimal yields. Inexpensive high-strength neodymium magnets offer a better option, resulting in higher yields with no detrimental effects on parasite viability.

## Background

Malaria parasites catabolize the proteinaceous component of haemoglobin as a nutrient source during their intracellular growth cycle in erythrocytes [1]. The haem moiety is not metabolized and accumulates in the parasite digestive vacuole. In order to reduce oxidative toxicity of the ferric iron, the excess haem is polymerized into the crystalline biomineral beta-haematin, more commonly referred to as haemozoin [1]. The haemozoin crystal becomes visible by microscopic examination during the trophozoite and schizont stages roughly halfway through the intraerythrocytic developmental cycle and continues to grow in size until the parasites are mature enough to egress from the erythrocyte.

The progressive accumulation of haemozoin throughout the life cycle has previously been exploited to preferentially isolate the late trophozoite and schizont stages. Due to the paramagnetic nature of haemozoin, passage of parasites over another paramagnetic material subjected to a strong magnetic field results in specific retention of late-stage parasites while allowing erythrocytes with no parasites or early-stage parasites to flow through. This approach was first reported over half a century ago using electromagnets to purify *Plasmodium vivax *parasites for use in vaccination efforts [2]. The problems of slow processing time and requirement for specialized equipment were subsequently circumvented through the use of 7,000 Gauss (G) permanent magnets [3], which were also employed for the direct purification of haemozoin [4]. More recently, commercial systems that are used for purification of immuno-labelled cells have been adapted for purification of parasite-infected red blood cells (iRBCs) [5,6] and haemozoin [7]. These systems have been demonstrated to work with many *Plasmodium *species and have been readily employed in field studies [5].

Despite the fact that magnetic purification of iRBCs has been in use for decades, a systematic study of the effects of varying the magnetic field strength has not been reported. This report demonstrates that magnetic purification of iRBCs and haemozoin can be accomplished using inexpensive and widely available rare-earth magnets with purity and yields that are superior to widely employed commercially available systems.

## Methods

### Parasite culture

*Plasmodium falciparum *W2 (MRA-157) was cultured using previously described methods [8]. Briefly, parasites were grown in human erythrocytes in RPMI 1640 media supplemented with 0.25 % Albumax II (GIBCO, Life Technologies, San Diego, California, United States), 2 g/L sodium bicarbonate, 0.1 mM hypoxanthine, 25 mM HEPES (pH 7.4), and 50 μg/L gentamycin, at 37°C, 5% O_2_, and 6% CO_2_. Parasites were loosely synchronized with a single round of sorbitol treatment prior to magnetic purification.

### Magnets

The following neodymium rare-earth magnets were purchased from Applied Magnets (Plano, TX): paired 1.5 in OD × 1.25 in ID × 0.75 in long × 90 degree grade N42 arcs (NA005), a diametrically magnetized grade N42 1 in diameter cylinder with a 0.25 in center hole (ND062-D), diametrically magnetized grade N42 1 in diameter cylinders (ND062-TD), and grade N52 1 in cubes (NB041). Magnetic fields were measured using a DC magnetometer (AlphaLab Inc., Salt Lake City, UT).

### Purification of haemozoin

Synchronized cultures at 2% haematocrit were grown to a ring parasitaemia of 10% followed by two additional days of culture to allow natural egress and release of haemozoin. Each LS column (Miltenyi Bioec, Auburn, CA) was loaded with 25 ml of resuspended culture, washed three times with 5 ml water, and eluted in 5 ml water. Haemozoin was recovered by centrifugation for 15 min at 12,500 × *g *and resuspended in 100 ul water. The final concentration of the recovered haemozoin was determined by dissolution into monomers and spectroscopic determination at 400 nm using a molar extinction coefficient of 10^5^, as previously described [9].

### Purification of iRBCs

Cultures were synchronized by one round of sorbitol synchronization and purified once the parasites reached the late-trophozoite stage. Each LD column (Miltenyi Biotec) was loaded with 50 ml of parasite culture at 2% haematocrit and 5-15% parasitaemia. Columns were washed once with 5 ml complete RPMI medium (RPMIc) and eluted in 5 ml RPMIc. Recovered cells were counted on a haemocytometer and purity was assessed by Giemsa-stained blood smear.

### Design and synthesis of magnetic purification stand

The purification stand was designed in SolidWorks 2009 (Dassault Systémes SolidWorks Corp., Concord, MA) as three separate components (body, lid, and legs). The body accommodates five 1 in diameter × 1 in length diametrically magnetized cylinders. Designs were exported as STL files and prototyped in acrylonitrile butadiene styrene (ABS+) plastic on a uPrint 3D printer (Dimension Inc., Eden Prairie, MN). The magnetic stand design files, appropriate for 3D printing, are available as supplementary files (Additional File 1). Updated versions are available in the 3D design repository at the DeRisi lab website [10].

### Statistical analysis

Percentages were arcsine transformed as summarized by Zar [11]. Means and confidence intervals were calculated on the arcsine-transformed data and converted back to linear space. All reported *p*-values are calculated from two-tailed *t*-tests assuming unequal variances on the arcsine-transformed data.

## Results and Discussion

### Purification of free haemozoin

Naturally generated haemozoin has traditionally been prepared through a series of extractions to remove all traces of contaminating nucleic acids, proteins, and lipids [12]. More recent studies examining the immuno-stimulatory properties of haemozoin have turned to the use of magnetic purification in order to isolate haemozoin in a state closer to its physiological form upon release from parasites undergoing egress from erythrocytes [7]. Although magnetic purification is increasingly employed in the field of malaria research, the relationship between magnetic field strength and haemozoin yield during magnetic purification procedures has not previously been examined.

**Figure 1:**
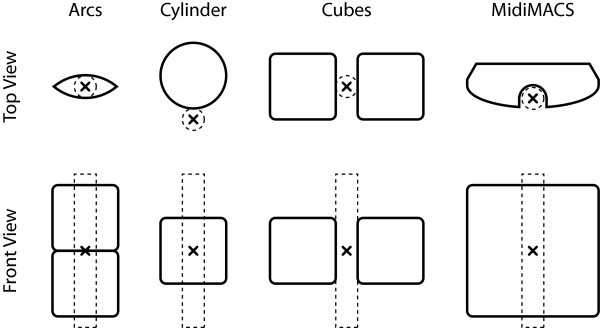
**Magnet configuration schematics**. Top and front views of the magnet configurations. Dark lines represent magnet outlines, dashed lines represent the purification columns, and "x" denotes the location of magnetic field measurement. Not drawn to scale.

Therefore, the performance of three configurations of permanent rare-earth neodymium magnets producing different strength fields was compared with a commercially available MidiMACS separator (Miltenyi Biotec). Purifications were performed on LS columns, which have a relatively fast flow rate that allows retention of haemozoin, but not late-stage parasites, as previously described [7]. For the weakest magnetic field, four arc-shaped motor magnets were used to create a two-inch long channel with a oval-shaped cross-section, resulting in a magnetic field of approximately 2,000 G (measured at the estimated centre position of the purification column; Figure 1). The MidiMACS separator possessed the third strongest field, measuring approximately 4,500 G. The second strongest field was generated by a diametrically magnetized cylinder (1 inch long × 1 inch diameter, with a ¼ inch hole down the centre), with a field of 5,100 G (estimated at a distance of one LS column radius from the surface). The strongest configuration was measured halfway between two one-inch cube magnets separated by a distance of one LS column diameter, measuring 8,400 G.

**Figure 2:**
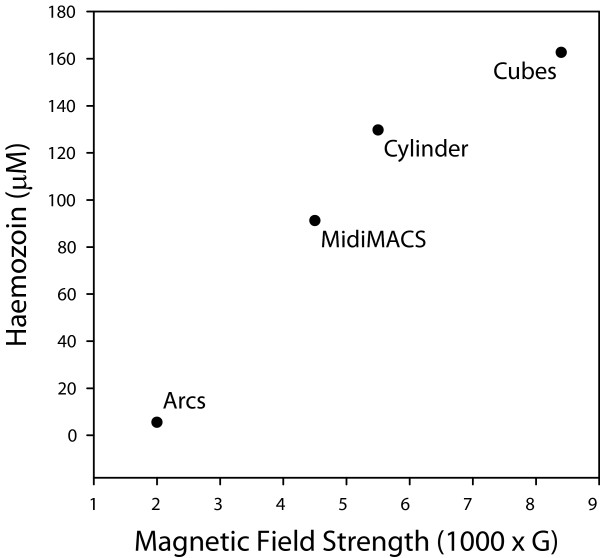
**Stronger magnetic fields result in higher haemozoin yields**. Haemozoin was purified on LS columns subjected to different magnet configurations.

Haemozoin was purified in parallel over LS columns in each magnet configuration and analysed for yield. The relationship between the measured magnetic field strength and concentration of the recovered haemozoin was roughly linear, with the strongest magnetic fields resulting in the highest haemozoin yields (Figure 2). The apparent deviation from linearity exhibited by the cube magnets suggests that further increases in the strength of the magnetic field may result in diminishing returns. Most importantly, the neodymium magnets provided considerably higher yields than the MidiMACS separator (42% more for the cylinder, 78% more for the cubes), which is the current standard in the field.

### Purification of infected erythrocytes

A previous study suggested that "better separation" of iRBCs might be achieved through the use of strong electromagnets [3]. However, it has remained unclear how different magnetic fields might influence the purity, yield, and viability of isolated iRBCs, especially in comparison with commercially available systems that are commonly used for this purpose. A comparison of iRBC purification on slow flow-rate LD columns (Miltenyi Biotec) subjected to the magnetic fields from the MidiMACS, cylinder magnet, and double cube magnet configurations was, therefore, undertaken.

Blood smears of the recovered iRBCs demonstrated high purity for all magnets (Table 1). The order of overall iRBC yield was similar to that for haemozoin, with cubes giving the highest yield and the MidiMACS separator giving the lowest yield. Notably, the yield from both the cylinder and cubes was considerably better than that from the MidiMACS separator (39% and 42% more than MidiMACS, respectively).

**Table 1:** Purification of iRBCs

Magnet	Field (G)	Purity (parasitaemia)	Parasites Recovered (% of input)
MidiMACS	4500	93.04%	7.9 × 10^7 ^(5.0%)
Cylinder	5100	92.77%	1.10 × 10^8 ^(7.0%)
2 × cubes	8400	92.52%	1.13 × 10^8 ^(7.2%)

### Practical application of magnetic purification

**Figure 3:**
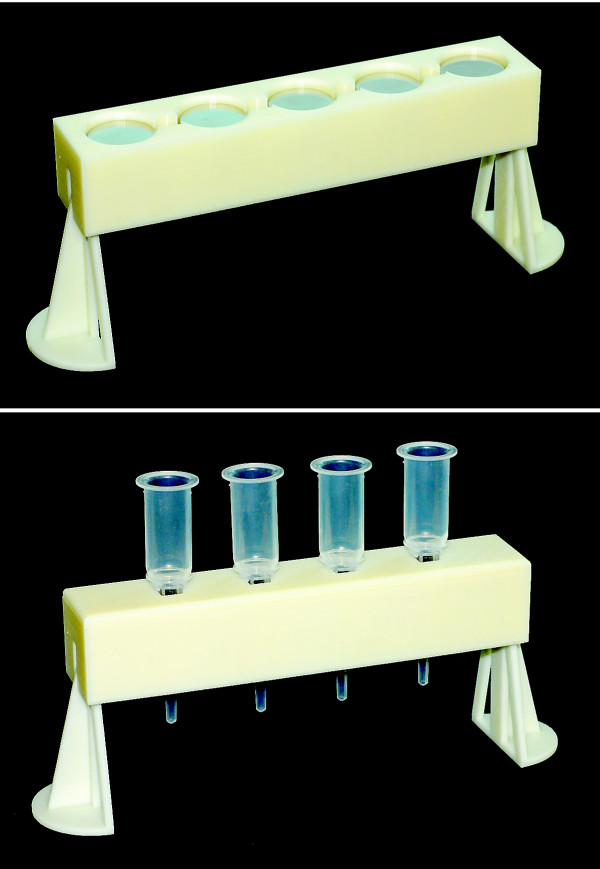
**Magnetic purification stand**. Photographs of the designed magnetic purification stand. (A) The stand with the lid removed showing the cylinder magnets. (B) The complete stand with four LS columns in place for purification. A disposable trough commonly used in high-throughput microplate applications fits beneath the column nozzles.

Although the two-cube configuration provided the highest yields in haemozoin and iRBC purifications, the cube magnets are considerably more difficult and dangerous to work with than the cylinder magnets as a result of having a stronger pull-force and no convenient surfaces for grasping when dislodging them from a surface. For practical application, a multiple diametrically-magnetized cylinder (ND062-TD) configuration, which generates magnetic fields that are almost as strong (7,500 G) as the pair of cubes used in previous experiments, was employed. A four-column stand was designed using CAD software, and a fully functional prototype was constructed on a 3D plastic printer (Figure 3). The design includes four column positions interdigitated with five cylinder magnets. Because the cylinders are able to rotate within their chambers, they automatically orient themselves when juxtaposed to adjacent magnets, which also maximizes the strength of the magnetic field at the column positions. The total cost of the system was approximately $40 for the cylinder magnets and $35 for the stand. The most recent versions of the CAD design files for these parts are freely available at the DeRisi lab 3D design repository [10].

Late stage parasites from synchronous cultures of *P. falciparum *were isolated in parallel using both the in-house-designed purification stand and three MidiMACS separators. The recovered parasites were assessed for overall yield using a haemocytometer, purity by blood smear, and viability by culture. The cylinder magnets recovered approximately 60% more parasites than the MidiMACS separators (25.9% and 15.0% total recoveries, respectively; *p *= 0.0002). Purity was high and comparable for both methods (cylinders 95.3% mean with 95% CI 93.6% to 96.9%; MidiMACS 95.7% mean with 95% CI 92.5% to 98.0%; *p *= 0.86).

**Figure 4:**
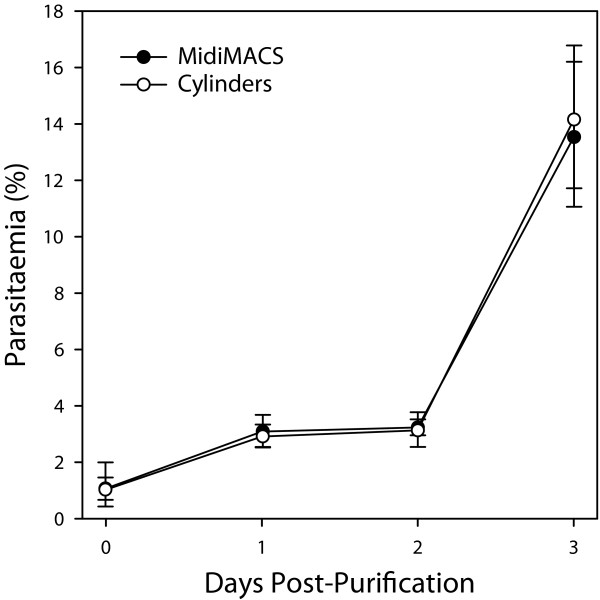
**Viability of purified parasites**. Purified parasites were diluted to 1% parasitaemia and cultured for 3 days after purification. Parasitaemia was monitored daily by blood smear. Means are depicted with error bars representing 95% confidence intervals for the purification stand (*n *= 4) and MidiMACS (*n *= 3) replicates. There were no statistically significant differences between the MidiMACS and cylinders at any time point.

In order to assess viability after being subjected to different strength magnetic fields, the purified parasites were diluted to 1% parasitaemia and monitored for growth over the next three days. The purified parasites from the two different preparations exhibited growth that was essentially identical, with a plateau between rounds of egress and reinvasion (Figure 4). These results indicate that the use of inexpensive high-strength neodymium magnets for malaria parasite purification results in significantly higher yields than commercial systems, with no adverse effects on parasite viability.

## Conclusions

Improvements to existing protocols for magnetic purification of free haemozoin and malaria-infected erythrocytes are described. In addition to giving higher yields of haemozoin and iRBCs with no detrimental effects on viability of purified parasites, the described systems are two orders of magnitude less expensive than commercially available cell purification magnets, making the approach much more widely accessible to the malaria research community.

## Competing interests

The authors declare that they have no competing interests.

## Authors' contributions

CCK designed and conducted experiments, designed the magnetic purification stand, and wrote the manuscript. EBW conducted experiments and contributed to preparation of the manuscript. JLD conceived the study and contributed to preparation of the manuscript. All authors read and approved the final manuscript.

## Supplementary Material

Additional file 1 **Magnetic purification stand design files**. A zip file containing three STL files corresponding to the body, lid, and legs of the purification stand. These files can be directly loaded into 3D printer software. Click here for file
